# Impact of photoreceptor density in a 3D simulation of panretinal laser photocoagulation

**DOI:** 10.1186/s12886-021-01945-z

**Published:** 2021-05-07

**Authors:** Kentaro Nishida, Shizuka Takahashi, Hirokazu Sakaguchi, Shigeru Sato, Masanori Kanai, Akihiko Shiraki, Taku Wakabayashi, Chikako Hara, Yoko Fukushima, Susumu Sakimoto, Kaori Sayanagi, Ryo Kawasaki, Kohji Nishida

**Affiliations:** 1 Department of Ophthalmology, Osaka University Graduate School of Medicine, 2-2 Yamadaoka, E-7, Suita, Osaka 565-0871 Japan; 2 Integrated Frontier Research for Medical Science Division, Institute for Open and Transdisciplinary Research Initiatives (OTRI), Osaka University, Suita, Osaka Japan

**Keywords:** Computer based methods, Panretinal laser photocoagulation, Photocoagulation index, Photoreceptor density, Photoreceptor destruction index

## Abstract

**Background:**

During panretinal photocoagulation (PRP), the outer retina, especially the photoreceptors, are destroyed. During such procedures, the impact of the retinal photocoagulation, which is performed in the same photocoagulated area, may change if it is applied to different locations with different photoreceptor densities. Thus, we aimed to evaluate the influence of photoreceptor density on PRP.

**Methods:**

We constructed a three-dimensional (3D) average distribution of photoreceptors with 3D computer-aided design (CAD) software using previously derived photoreceptor density data and calculated the number of photoreceptors destroyed by scatter PRP and full-scatter PRP (size 400-μm on the retina, spacing 1.0 spot) using a geometry-based simulation. To investigate the impact of photoreceptor density on PRP, we calculated the ratio of the number of photoreceptors destroyed to the total number of photoreceptors, termed the photoreceptor destruction index.

**Results:**

In this 3D simulation, the total number of photoreceptors was 96,571,900. The total number of photoreceptors destroyed by scatter PRP and full-scatter PRP were 15,608,200 and 19,120,600, respectively, and the respective photoreceptor destruction indexes were 16.2 and 19.8%, respectively.

**Conclusions:**

Scatter PRP is expected to have 4/5 of the number of photoreceptors destroyed by full-scatter PRP.

**Supplementary Information:**

The online version contains supplementary material available at 10.1186/s12886-021-01945-z.

## Background

Retinal ischemia is the reduction or cut-off of the retinal blood supply, which can result in visual impairment and blindness [1]. Severe retinal ischemic diseases include proliferative diabetic retinopathy (PDR) and central retinal vein occlusion (CRVO). PDR is caused by the progression of diabetic retinopathy, a microvascular complication of diabetes, and accounts for one-third of the rubeosis iridis cases [2]. CRVO is caused by a circulatory disturbance at the trunk of the central retinal vein near the lamina cribrosa and accounts for neovascular glaucoma (NVG) [3] and 28% of all cases with rubeosis iridis [4].

Severe ischemia causes dysfunction and destruction of photoreceptors and the production of cytokines [5], such as vascular endothelial growth factor (VEGF) [6]. These conditions cause leakage in the retinal vessels and neovascularization, leading to proliferative retinopathy. Panretinal laser photocoagulation (PRP) is the gold standard treatment for severe retinal ischemic diseases, including PDR [7–10] and rubeosis iridis, which arise from ischemic CRVO [11, 12]. There is strong evidence that PRP prevents the development of NVG in PDR [7]. Prompt PRP also prevents the development of NVG in ischemic CRVO eyes with a neovascular 2’clock iris/angle [12]. However, the mechanisms underlying the effects of PRP are not fully understood. Various potential mechanisms have been suggested [5], including the destruction of photoreceptors, greater oxygen supply from the choroid to the inner retina [13–17], and the destruction of retinal neurons that produce cytokines [5]. It has been postulated that the effects of the destruction of photoreceptors may be related to the fact that they require large quantities of oxygen and produce large amounts of VEGF during ischemic conditions [6].

The influence of retinal photocoagulation, which is performed in the same photocoagulated area, may change when retinal photocoagulation is applied to the different regions with different photoreceptor densities. The current protocol for PRP, typically followed by ophthalmologists, involves a 400-μm spot on the retina, and a recommended 100-ms pulse. The intensity of the laser should cause mild white retinal burns 1 spot width apart [18]. PRP can be performed using a scatter (to the equator) [19] or full-scatter (to the ora serrata) pattern, depending on the retinal conditions. The protocol determines the total area of photocoagulated retinal lesions. In a previous study, we calculated this area using geometric methods and calculated the photocoagulation index [20, 21], which refers to the ratio of the total retinal photocoagulated lesion area to the whole retina, for full-scatter PRP and scatter PRP. This was the first attempt to quantitatively compare full-scatter PRP with scatter PRP [20]. However, that study did not consider photoreceptor density.

In the present study, we constructed a three-dimensional (3D) average distribution of photoreceptors with a 3D computer-aided design (CAD) software (Solidwork 2017 standard®; Dassault Systèmes SolidWorks Corporation, Waltham, MA, USA, https://www.solidworks.com/) using data from a previous photoreceptor density study [22] and calculated the number of photoreceptors destroyed by scatter PRP and full-scatter PRP using our geometry-based method. We also utilized the concept of the photoreceptor destruction index, which is the ratio of the number of photoreceptors destroyed to the total number of photoreceptors. Both scatter PRP and full-scatter PRP were simulated to investigate the impact of photoreceptor density on PRP.

## Methods

### 3D average distribution of photoreceptors

We constructed the 3D average distribution of photoreceptors using 3D CAD software (SolidWorks 2017 standard®) using the photoreceptor density data from a previous study [22]. Four graphs of photoreceptor densities in four directions (superior, inferior, nasal, and temporal) were obtained and representative graphs with the same eccentricity of the fovea were averaged, and an average photoreceptor density graph was created. This graph was rotated around the y-axis, and the 3D average distribution of photoreceptors was constructed using Solidwork 2017 standard®. In addition to the island-shaped solid representing the total photoreceptor distribution, the cubic volume of the solid represents the number of photoreceptors.

### Simulation of PRP

Simulations of scatter PRP and full scatter PRP (size 400 μm on the retina, 1 spot width apart) were performed using a geometry-based simulation as previously described [20, 21]. To facilitate a better understanding of some relevant concepts, we describe important aspects of the geometry-based simulation as follows.

**Fig. 1:**
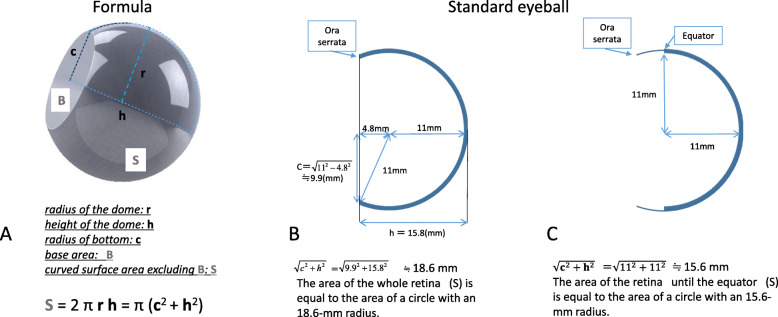
Geometric formula to calculate the curved surface area of a spherical dome and anatomical dimensions of a standard eyeball. **a**. If the radius of the dome is **r**, the height of the dome is **h**, the radius of the bottom is **c,** and the base area is **B**, the curved surface area (**S**), excluding **B** of the dome is **S** = 2π**rh** = π (**c**^2^ + **h**^2^). **b**. The area of the whole retina and the retina up to the equator. These dimensions were derived from a textbook [23]. **c**. The areas in S are equal to the areas of circles with radii of 18.6 mm and 15.6 mm, calculated via the formula presented in (**a**)

We used a geometric formula to calculate the curved surface area of a spherical dome (a portion of a sphere transected by a plane; see Fig. 1a). If the radius of the dome is **r**, the height of the dome is **h**, the radius of the base is **c**, and the area of the base is **B**, the curved surface area (**S**) excluding **B** of the dome is as follows (see Fig. 1a):

$$ \mathbf{S}=2\pi \mathbf{rh}=\pi\ \left({\mathbf{c}}^2+{\mathbf{h}}^2\right) $$

If the values of **c** and **h** for the eye are known, the curved area of the retina as a whole is equal to the area of a circle with a radius of $\sqrt{{\mathbf{c}}^2+{\mathbf{h}}^2}$. The area of the whole retina can be considered to be a part of a sphere; thus, the entire retinal area corresponds to the curved surface area of a spherical dome. The dimensions of a standard eye were derived from a textbook [23] (Fig. 1b), and inserting these values into the abovementioned equation shows that the whole retinal area is equal to the area of a circle with a radius of 18.6-mm (Fig. 1b).

**Fig. 2:**
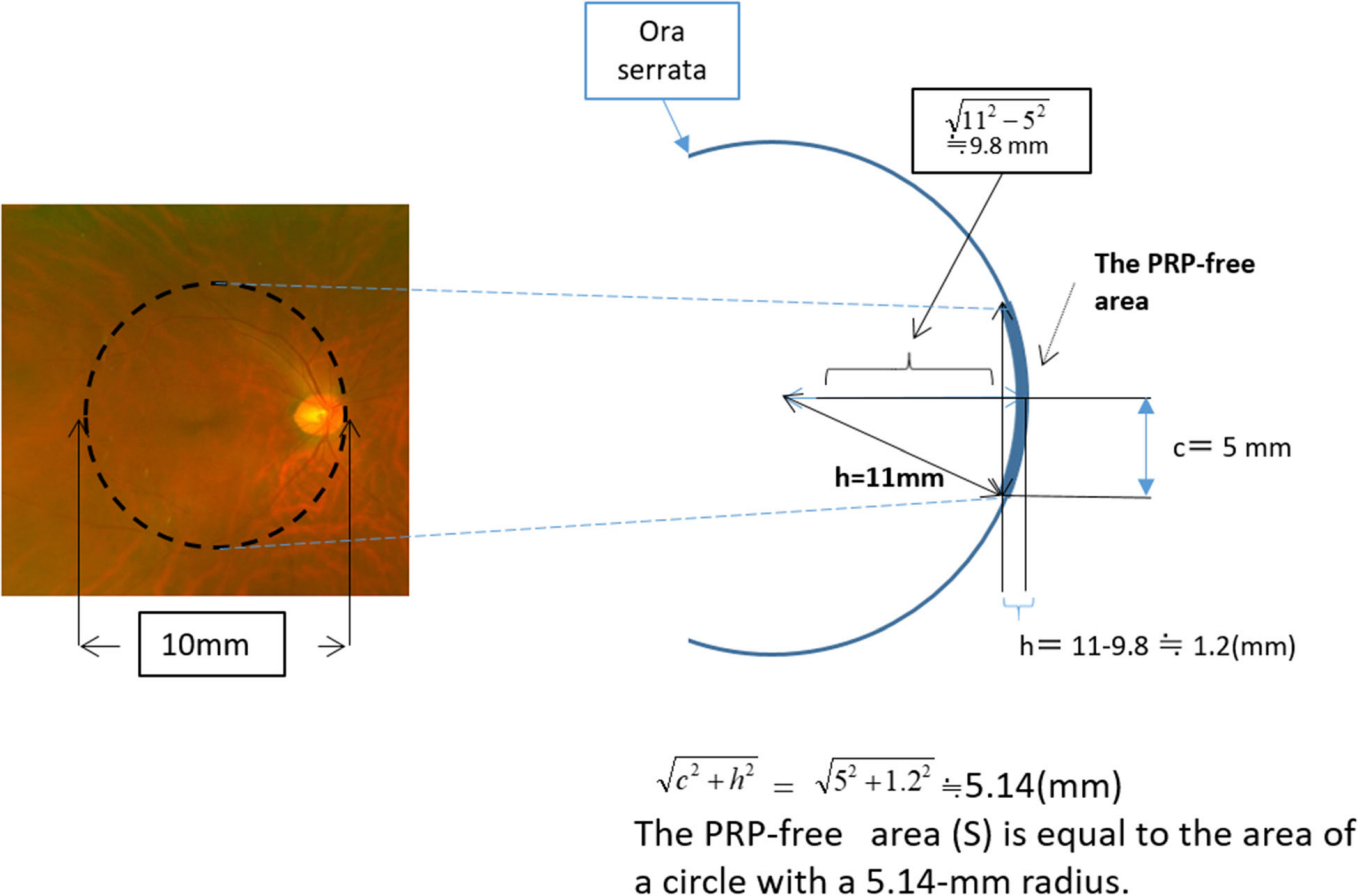
The PRP-free area. The anatomical dimensions of a standard eyeball taken from a textbook [23]. The PRP-free area was set as a circle with a radius of 5 mm (broken line). The PRP-free area is equal to the area of a circle with a 5.14-mm radius calculated using the formula presented above in Fig. 1a

The scatter PRP normally stops at the retinal equator. The retinal area up to the equator corresponds to the area of a circle with a radius of 15.6 mm (Fig. 1c). PRP was conducted without photocoagulation of the vascular arcade region and the optic disc. We set the PRP-free area as a circle with a radius of 5 mm, with a surface area equal to the area of a circle with a radius of 5.14 mm (Fig. 2). We assumed that the size of the spot on the retina was 400 μm, and that the spacing between each circle was equivalent to one spot.

To simulate the scatter PRP and full-scatter PRP based on photoreceptor densities, we used Solidwork 2017 standard® to draw circles of the same diameter (400 μm) with a distance of 400 μm between each circle. We then drew three concentric circles with radii of 18.6 mm (Fig. 1b), 15.6 mm (Fig. 1c), and 5.14 mm (Fig. 2) on the bottom and center of the island-shaped solid that represented the photoreceptor distribution. Homothetic diagrams were drawn using Solidwork 2017 standard®. For example, in the scatter PRP simulation, we kept the spots inside the 15.6-mm radius circle and outside the 5.14-mm radius circle. Similarly, in the full-scatter PRP simulation we kept the spots inside the 18.6-mm radius circle and outside the 5.14-mm radius circle. Spots on the borders of these circles were excluded from both simulations. Finally, these circles were punched out at a right angle from the bottom of the island-shaped solid, and we calculated the volume of the island-shaped solid and translated it into the number of photoreceptors. The photoreceptor destruction index was also calculated for comparison.

## Results

**Fig. 3:**
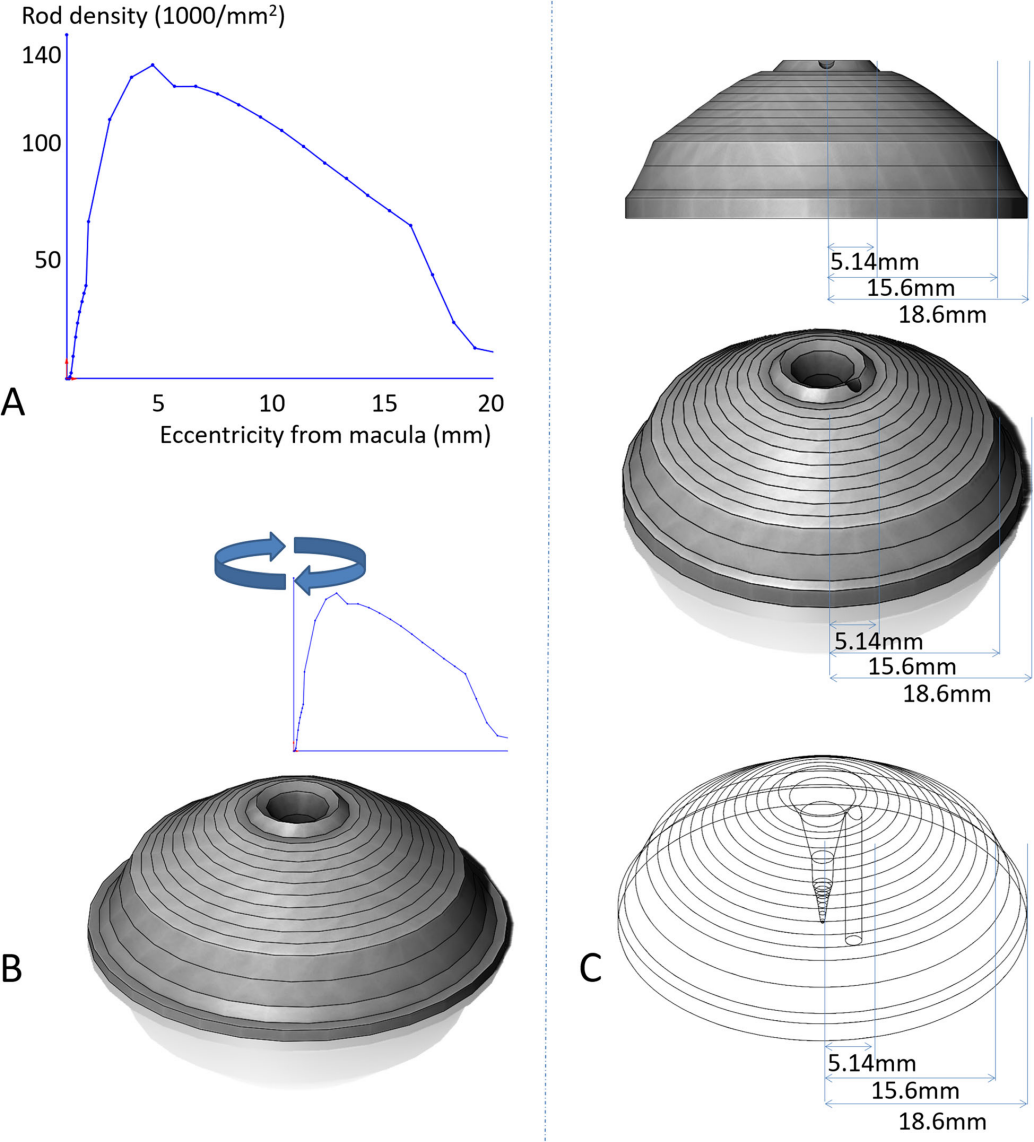
The average photoreceptor density graph and the 3D average distribution of photoreceptors. **a** The average photoreceptor density graph was created using photoreceptor density data from a previous study [22]. **b** This graph was rotated around the y-axis. **c** A circular cylinder corresponding to the optic disc was hollowed out, and the 3D average distribution of photoreceptors was constructed

An average photoreceptor density graph was created using photoreceptor density data from a previous study [22] (Fig. 3a). This graph was rotated around the y-axis (Fig. 3b) and we created an island-shaped 3D average distribution of photoreceptors (Fig. 3c, Additional file 1) with a volume of 9657.19 mm^3^. This volume was translated into the corresponding number of photoreceptors, which were, 96,571,900 in number.

**Fig. 4:**
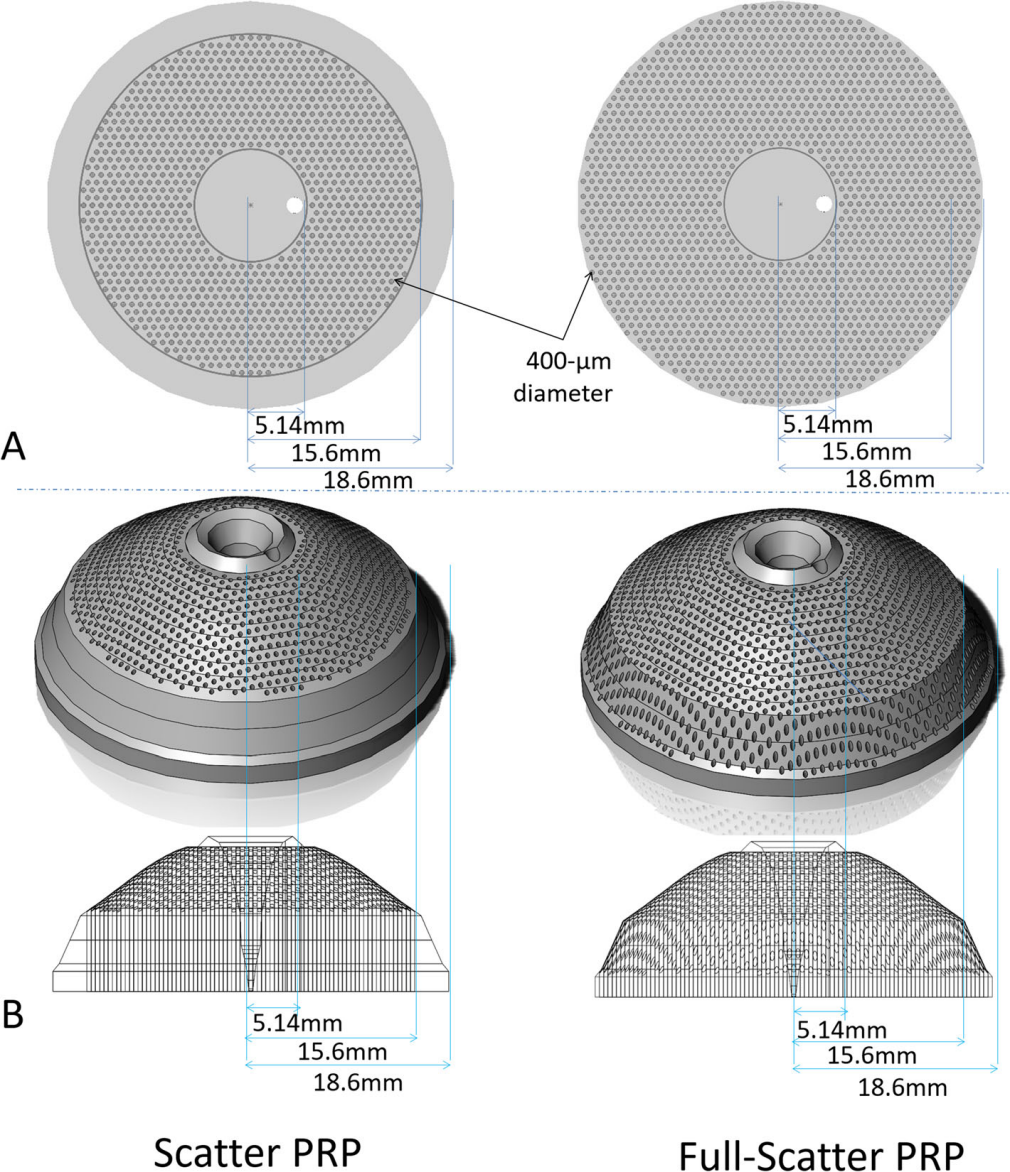
Simulations of scatter PRP and full-scatter PRP using a geometry-based simulation and based on photoreceptor densities. **a** Simulations of scatter PRP and full-scatter PRP (size 400 μm on the retina, 1 spot width apart) using a geometry-based simulation. **b** Simulation of scatter PRP and full-scatter PRP based on photoreceptor densities. The numbers of photoreceptors destroyed and the photoreceptor destruction indexes are shown in Table 1

The numbers of spots derived from scatter PRP and full-scatter PRP were 1261 and 1837, respectively (Fig. 4a) [20]. The volumes of the island-shaped solids that were punched out by scatter PRP and full-scatter PRP were 8096.37 mm^3^ and 7745.13 mm^3^, respectively. Furthermore, the total numbers of residual photoreceptors after scatter PRP and full-scatter PRP were 80,963,700 and 77,451,300, respectively (Fig. 4b). In addition, the total numbers of photoreceptors destroyed by scatter PRP and full-scatter PRP were 15,608,200 and 19,120,600, respectively. The photoreceptor destruction indexes for scatter PRP and full-scatter PRP were 16.2 and 19.8%, respectively (Table 1).

**Table 1:** The number of photoreceptors destroyed, photoreceptor destruction indexes, and photocoagulation indexes

	Before PRP	Scatter PRP	Full-scatter PRP
Total number of residual photoreceptors	96,571,900	80,963,700	77,451,300
Total number of destroyed photoreceptors	0	15,608,200	19,120,600
**Photoreceptor destruction index (%)**	0	16.2	19.8
**Photocoagulation index (%)**	0	14.3	21.3

## Discussion

We simulated PRP based on the photoreceptor density using a 3D CAD software. This is the first report of a study involving a simultaneous investigation of both PRP and photoreceptor density.

The numbers of indexes between the simulations were similar when comparing the two-dimensional simulation in our previous study [20] was compared with the results of the present simulation base on photoreceptor densities (Table 1). However, when using this photoreceptor density-based simulation, the difference between the scatter PRP and full-scatter PRP decreased. Additionally, the number of photoreceptors in the peripheral area was overestimated in this photoreceptor density-based simulation; the difference between scatter PRP and full-scatter PRP is likely to be smaller in real-world scenarios. This result suggests that when we treat patients with severe ischemia, such as patients with open-angle NVG [3, 24, 25], the impact of adding full-scatter PRP to scatter PRP is small because the additional treatment only increases the photoreceptor destruction index by 3.6%. In the context of photoreceptor destruction, the impact of peripheral PRP may be limited, but the loss of visual function [26, 27] from additional PRP is also limited. If peripheral PRP is not performed thoroughly, additional peripheral PRP remains an option for the treatment of NVG [11].

Some patients who have already undergone a well-performed full-scatter PRP still exhibit an open-angle refractory NVG. In this case, anti-VEGF therapy is effective [28–30]; however, this is not a fundamental therapeutic method, but a symptomatic therapy [31, 32], and additional PRP should be considered. Furthermore, it is difficult to determine where to perform additional PRP when full-scatter PRP has already been well performed. In such cases, additional scatter PRP can be performed. This is because if scatter PRP is performed in the residual area that is not photocoagulated (for example, additional scatter PRP with 1-spot spacing) the photoreceptor destruction index will be expected to increase by an additional 16.2%, based on the model. Another option is additional PRP inside the vascular arcade. Thirty-nine spots were needed for one additional row of photocoagulation inside the vascular arcade (data not shown); the photoreceptor destruction index is expected to increase by an additional 0.7% (data not shown). This is one-fifth of the impact of the additional full-scatter PRP to scatter PRP. These approaches are suggested in the context of photoreceptor destruction, and additional clinical studies are needed for clinical validation.

The current study had some limitations and one of them was our 3D model of photoreceptor distribution. We created a graph of the average of the photoreceptor density because a 3D model of photoreceptor distribution was not available. However, a previous study [22] reported that the number of photoreceptors in many parts of the retina in a cadaveric eye was counted precisely and the distribution of rods in each retina was simulated. Therefore, the data used were only those of one cadaveric eye, and thus, the variability was not taken into consideration. Indeed, there are some variations in peak density, quadrant of the peak, the eccentricity of the peak, the total number of rods, and the mean density of rods [22]. However, we compared the number of destroyed photoreceptors of scatter PRP with that of full-scatter PRP. Some variations in peak density, quadrant of the peak, and eccentricity of the peak were compensated for because the peak of the rod density is inside the equator. Hence, it does not affect the number of destroyed photoreceptors of the scatter PRP and full-scatter PRP. There are some variations in the total number and mean density, which is related to the total number. However, we evaluated the difference using indexes, and these variations were compensated for. The variation in the density of rod photoreceptors in the peripheral retina may have affected the quantitative evaluation of our simulation. Nevertheless, the density of rod photoreceptors in the peripheral retina was low, and the effect of the variation in the density of rod photoreceptors in the peripheral retina in our simulation was negligible.

Second, our 3D model of photoreceptor distribution showed concentric distribution of rods; however, in actual clinical settings, this distribution is not concentric [22, 33, 34], but differences in the distribution of rods at the same distance from the macula in four directions are all within 15% of each other; most are within 10%. The influence of the difference in the distribution of the rod was limited by using the volume of the island-shaped solid created by the average photoreceptor density graph. Additionally, the regions of the PRP in this simulation were concentric, which further limited the influence of the difference in the distribution of the rods. Notably, the total number of rod photoreceptors was 96,571,900 in this photoreceptor density-based simulation. This is quite similar to the average total number of rod photoreceptors which were 92,000,000 (range 77,900,000–1,073,000) in a previous report [22] from which the simulation data were obtained. Thus, our results have some validity. In the current study, we did not count the cone photoreceptors because the number of cones is 10–60 times lower than that of rod photoreceptors in the area [22] in which PRP was performed.

Lastly. Our study did not incorporate adjustments for axial length or atrophic creep [35]. To the best of our knowledge, no definitive study has determined the distribution of photoreceptors at various axial lengths. It is worth noting that, a simulation base on “normal axial length” may be applicable to the majority of people. Atrophic creep is related to the strength of the laser burn and the thickness of the retina, but its size cannot be predicted [35, 36]. Simulations incorporating those factors would be much more complex, and in the current study we used standard eye parameters and ideal laser burns without atrophic creeps.

Although additional studies are needed to evaluate the efficacy of photoreceptor destruction indexes clinically, this is the first attempt to simulate the number of photoreceptors destroyed by photocoagulation therapy. This may then lead us to quantitatively evaluate photocoagulation therapy and provide a new photocoagulation strategy, especially for NVG in the future.

## Conclusions

According to our 3D simulation, scatter PRP is expected to have 4/5 of the number of photoreceptors destroyed by full-scatter PRP.

## Supplementary Information

**Additional file 1.**

## Data Availability

All the data supporting our findings are available through email request from the corresponding author.

## Abbreviations

VEGF: Vascular endothelial growth factor
PRP: Panretinal laser photocoagulation
3D: Three-dimensional
CAD: Computer-aided design
PDR: Proliferative diabetic retinopathy
CRVO: Central retinal vein occlusion
NVG: Neovascular glaucoma
